# Fetal Gallstones in a Newborn after Maternal COVID-19 Infection

**DOI:** 10.1155/2021/3688173

**Published:** 2021-11-18

**Authors:** Gurleen Kaur Kahlon, Anna Zylak, Patrick Leblanc, Noah Kondamudi

**Affiliations:** Department of Pediatrics, The Brooklyn Hospital Center, 121 Dekalb Avenue, Brooklyn, NY 11201, USA

## Abstract

Fetal gallstones are rare incidental findings on ultrasound during pregnancy. We describe a newborn girl with gallstones that was born to a mother who had COVID-19 infection during her last trimester. The baby remained asymptomatic, and the stones resolved spontaneously without any treatment or complications within six weeks of birth. Several conditions predispose to fetal gallstones, and it is unclear if the recent maternal COVID-19 infection had any role in the occurrence of these abnormalities or was merely coincidental. This is the first case describing an association of fetal gallstones with a COVID-19 infection in pregnancy.

## 1. Introduction

Fetal gallstones or sludge are uncommon and are usually an incidental sonographic finding during pregnancy [1]. There have been no reports of any association between maternal COVID-19 infection and fetal gallbladder abnormalities. COVID-19 infection can affect the gastrointestinal system, and in one study, 54% of patients with right upper quadrant ultrasound revealed a dilated sludge-filled gallbladder suggestive of biliary stasis [2]. In another report, SARS COVID-2 virus causing COVID-19 has been detected in the bile of a patient with gallbladder disease [3]. Gallbladder wall edema has been described in children affected with COVID-19-associated multisystem inflammatory syndrome [4]. Sileo et al. reported gallbladder calcification in a 38-week gestation newborn whose mother was diagnosed with COVID-19 infection at 35 weeks of gestation [5]. Presently, there is insufficient data regarding the effects of COVID-19 illness on maternal, perinatal, and neonatal outcomes. It is unclear if maternal COVID-19 infection has any role in the occurrence of gallbladder abnormalities [6].

## 2. Case Presentation

**Figure 1:**
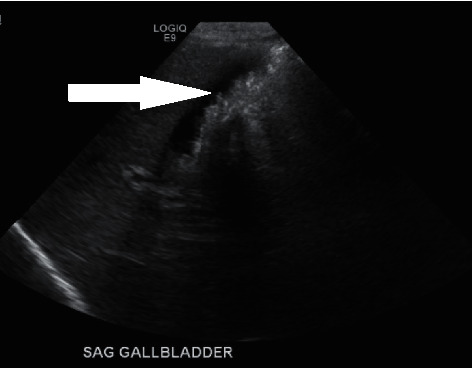
Biliary sludge in the dependent aspect of the gallbladder.

**Figure 2:**
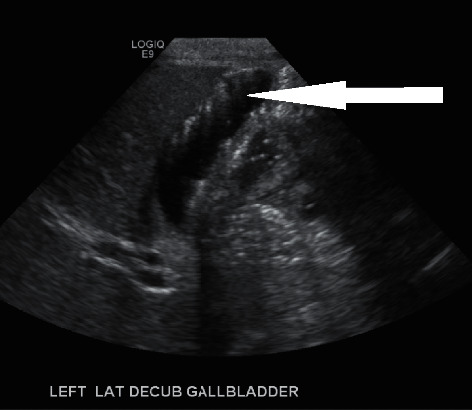
Biliary sludge and gravel of stones.

**Figure 3:**
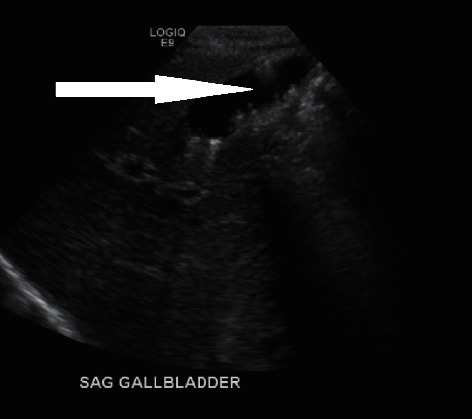
Multiple gallstones.

**Figure 4:**
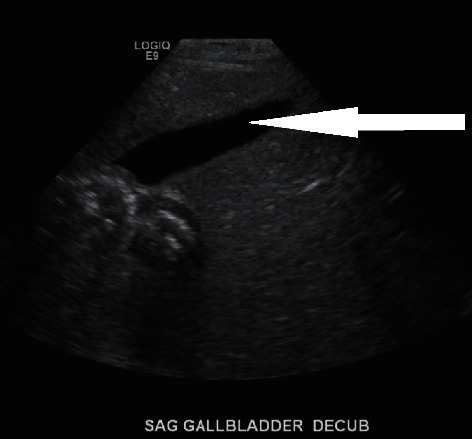
Normal gallbladder with complete resolution of the initial finding.

A 34-year-old obese G4 P1021 woman with a BMI of 32 was admitted at 38 weeks of gestation to the antenatal unit for a scheduled repeat cesarean section. Her past medical history was significant for an unspecified cardiac rhythm abnormality and well-controlled chronic hypertension on metoprolol 50 mg twice a day. The family history was significant for high cholesterol, diabetes, hypertension, gastric ulcers, cardiovascular disorders, and cancers of the breast and vagina. She initially received prenatal care at another facility and was transferred to our hospital during her last trimester. The patient reported that she developed a sore throat, cough, and body pain six weeks before and was diagnosed at the local emergency department with COVID-19 illness (PCR-positive). The patient reported that a sonogram done 3 weeks before showed a possible gallbladder abnormality. She was advised supportive care and self-quarantine for two weeks, and since then, she has made a full recovery. All prenatal lab outcomes (RPR, Hepatitis B surface antigen, HIV, QuantiFERON, Group B *Streptococcus*, and repeat COVID-19 PCR) were negative upon the present admission. The mother received cefazolin preoperatively and underwent an uneventful cesarean section. A healthy baby girl was delivered with APGAR scores of nine and nine at 1 min and 5 min, respectively. The amniotic fluid was clear with artificial rupture of membranes 2 minutes before delivery. The umbilical cord showed three vessels. The mother had a blood group of B Rh-negative, the baby's blood group was O Rh-positive, and DAT was negative. Due to Rh incompatibility, the mother received 1 gram of RhoGam postpartum. On physical examination, the baby was pink, alert, and active. Her anthropometric measurements were as follows: birthweight was 3227 grams (67th percentile), length was 48.26 cm (47th percentile), and head circumference was 35 cm (85th percentile). The vital signs were within the normal range for age at birth. The patient's skin exam was remarkable for erythema toxicum predominantly on the face and a lumbosacral Mongolian spot. Throughout her hospital stay, she had no visible jaundice, abdominal distention, or palpable masses. She was voiding and stooling normally. At 24 hours of life, direct bilirubin was 0.2 mg/dl, total serum bilirubin was 4.4 mg/dl, and alkaline phosphatase was 99 U/L, all within the normal range. Due to the maternal concern surrounding the previous sonogram, an abdominal ultrasound was performed soon after birth (Figures 1–3) which showed gravel of stones and sludge in the dependent aspect of the gallbladder. There was no associated gallbladder wall thickening or pericholecystic fluid to suggest acute cholecystitis and no dilatation of the intrahepatic or extrahepatic biliary ducts. The patient was clinically stable without any evidence of hyperbilirubinemia and was discharged home on the day of life two with close outpatient follow-up. At the two-week follow-up, a cardiac murmur was detected attributing to hemodynamically insignificant peripheral pulmonary stenosis. At the six-week follow-up, there was complete resolution of the gallbladder abnormalities (Figure 4).

## 3. Discussion

The incidence of fetal gallstones of sludge can range from 5 per 1000 to 1 in 3000 live births, and the actual prevalence is estimated to be around 0.5 to 0.7/10,000 live births [7]. The frequency of diagnosis has been increasing recently due to the more ubiquitous use of ultrasound examinations. Fetal gallstones play no role in fetal or postnatal prognosis and resolve spontaneously after birth without any need for intervention [8]. The exact pathogenesis of fetal gallstones remains unknown. Various factors (Table 1) have been implicated that potentially have a role in the formation of gallstones [9–13]. There have been no previous reports regarding the association of fetal gallstones with COVID-19 infection. While it is possible that conditions listed in the table, particularly obesity, may have played a role, it is also conceivable that maternal COVID-19 infection played a significant part in the occurrence of these stones. Bhayana et al. [2] demonstrated a dilated sludge-filled gallbladder suggestive of biliary stasis in 54% of their cases. While the exact mechanism of this association remains unexplained, biliary stasis does predispose to gallstone formation.

The fetal gallbladder starts developing around the 4th week of gestation from the embryonic foregut [14]. The fetal gallbladder is commonly an oval or teardrop-shaped structure and has no active role in fetal gastrointestinal physiology [15]. The fetal gallbladder is visible on ultrasound as early as the second trimester in the form of an elliptical hypoechoic or anechogenic body, present to the right of the intrahepatic umbilical vein [16]. Because the umbilical vein and fetal gallbladder have a similar ultrasonographic appearance, prior identification of the umbilical vein is recommended. Fetal gallstones manifest as echogenic foci with acoustic shadowing compared to gallbladder sludge, which is hyperechoic without any acoustic shadowing. Gallbladder sludge is considered a precursor of fetal gallstones and is reported to occur in approximately 40% of fetal gallstone cases. It is considered a more frequent finding compared to fetal gallstones. Fetal gallstones and gallbladder sludge are mainly seen during the third trimester [11, 13, 16]. The mechanisms postulated for the occurrence of gallbladder sludge or fetal gallstones are twofold. It may be due to hematoma in the maternal placenta, which results in hemoglobin breakdown leading to bilirubin formation and eventually gallstones. The other mechanism may be related to increased maternal estrogen levels, which can depress bile acid synthesis and increase cholesterol secretion, eventually leading to gallstone formation [1, 8]. Most infants found to have gallbladder stones/biliary sludge remain asymptomatic, and the abnormalities resolve spontaneously with time. One study involving 63 infants with fetal cholelithiasis and followed with serial ultrasound examinations found that complete resolution occurred in 70% of cases within two months and greater than 90% of cases within six months. Only two patients had persistent fetal cholelithiasis beyond 12 months. Schwab et al. followed a subset of 17 patients for a 3–20-year duration and found no complications or sequelae related to fetal gallstones [11, 17]. Therapeutic interventions described in the literature include the use of ursodeoxycholic acid and laparoscopic cholecystectomy. There is presently no evidence that therapeutic interventions are superior to observations awaiting spontaneous resolution. Medical treatment or cholecystectomy should be considered for rare persistent cases, symptomatic cases, or those with related complications [9–11, 13, 18]. There appears to be no difference in resolution time between sonogram-detected fetal gallstones and biliary sludge detection during pregnancy, and both scenarios have no impact on the obstetrical management or the delivery process [1, 9, 11]. Persistent fetal cholelithiasis/biliary sludge beyond one year of age may indicate a worse prognosis [19]. Associated anomalies can influence prognosis. These are reported in 20–30% of cases and include primary pulmonary hypertension, hydronephrosis, renal ectopia, hemivertebra syndrome, and hydrocephalus. Thus, for patients identified prenatally with gallstones and biliary sludge, a careful evaluation to detect associated abnormalities is prudent [12, 20].

## 4. Summary

We report an asymptomatic newborn with gallstones and biliary sludge detected on abdominal ultrasound soon after birth, which was entirely resolved by six weeks of age. It is unclear if the recent maternal COVID-19 infection had any role in the occurrence of these abnormalities or was merely coincidental. Fetal gallstones are rare and mostly resolve without any medical or surgical intervention.

## Figures and Tables

**Table 1:** Maternal and fetal causes associated with fetal cholelithiasis [4, 9–12].

Maternal causes	Fetal causes
(1) Hemolytic diseases (spherocytosis, sickle cell anemia, thalassemia)	(1) Hemolytic diseases (spherocytosis, sickle cell anemia, thalassemia)
(2) History of cholelithiasis	(2) Congenital malformation (CVS, GI, urologic, and skeletal)
(3) Intrahepatic cholestasis of pregnancy	(3) Anomalies of biliary tract or biliary obstruction
(4) Intestinal malabsorption	(4) Chromosome anomalies (trisomy 21; translocation 10, 11)
(5) Chronic liver disease	(5) Congenital malabsorption syndrome
(6) Hypercholesterolemia	(6) Pancreatic cystic fibrosis
(7) Increased estrogen and progesterone levels	(7) IUGR
(8) All types of diabetes	(8) Fetal obesity or macrosomia
(9) Obesity	(9) Oligohydramnios
(10) Narcotic use (methadone)	(10) Polyhydramnios
(11) Ceftriaxone treatment	(11) Prematurity
(12) Anticancer drug treatment	(12) Prenatal leukemoid reaction
(13) Prostaglandin E2 treatment	(13) Hepatitis
(14) Furosemide treatment	(14) Fetal- maternal blood group incompatibility (rhesus or ABO blood group incompatibility)
(15) Prolonged fasting	(15) Idiopathic
(16) Dehydration	
(17) Enteral nutrition	
(18) Intoxication with denatured oil treated with steroid	
(19) Twin pregnancy	
(20) Twin pregnancy with fetal demise of one twin	
(21) Placental abruption	
(22) Sepsis	

## Data Availability

No data were used to support this study.
